# Social accountability and nursing education in South Africa

**DOI:** 10.3402/gha.v8.27879

**Published:** 2015-05-11

**Authors:** Susan J. Armstrong, Laetitia C. Rispel

**Affiliations:** 1 Centre for Health Policy & Medical Research Council Health Policy Research Group, School of Public Health, Faculty of Health Sciences, University of the Witwatersrand, Johannesburg, South Africa; 2 Department of Nursing Education, School of Therapeutic Sciences, Faculty of Health Sciences, University of the Witwatersrand, South Africa

**Keywords:** nursing education, social accountability, nurses, transformative education, South Africa

## Abstract

**Background:**

There is global emphasis on transforming health workforce education in support of universal health coverage.

**Objective:**

This paper uses a social accountability framework, specifically the World Health Organization's six building blocks for transformative education, to explore key informants’ perspectives on nursing education in South Africa.

**Methods:**

Using a snowballing sampling technique, 44 key informants were selected purposively on the basis of their expertise or knowledge of the research area. Semi-structured interviews were conducted with the key informants after informed consent had been obtained. The interviews were analysed using template analysis.

**Results:**

South Africa has strategic plans on human resources for health and nursing education, training, and practice and has a well-established system of regulation and accreditation of nursing education through the South African Nursing Council (SANC). Key informants criticised the following: the lack of national staffing norms; sub-optimal governance by both the SANC and the Department of Health; outdated curricula that are unresponsive to population and health system needs; lack of preparedness of nurse educators; and the unsuitability of the majority of nursing students. These problems are exacerbated by a perceived lack of prioritisation of nursing, resource constraints in both the nursing education institutions and the health training facilities, and general implementation inertia.

**Conclusion:**

Social accountability, which is an essential component of transformative education, necessitates that attention be paid to the issues of governance, responsive curricula, educator preparedness, and appropriate student recruitment and selection.

As the largest single group of health-care providers in any country, nurses have the potential to bridge the gap between communities and the health-care system, coordinate care for patients with increasingly complex disease profiles, and accelerate the achievement of universal health coverage (1–4). However, the Institute of Medicine in the United States has pointed out that such major health system changes will require equally profound changes in the education of nurses to prepare them for new and transformed roles and responsibilities (4).

Around the world, the discourse on the education of health professionals has intensified, focusing on the need to increase numbers and to improve the quality, relevance, and retention of graduates (3, 5–8). However, the call for the scale-up of education programmes has been accompanied by a clear enunciation of systemic challenges in health professional education (5, 8). These challenges include the following: the mismatch between professional competencies and patient and population health priorities; poor teamwork; insufficient emphasis on primary health care (PHC); maldistribution of health-care professionals; and weak stewardship and leadership to improve health system performance (5, 8).

Nonetheless, there is a convergence of ideas that these challenges could be overcome through the transformative scale-up of medical, nursing, and midwifery education (3–8). Critical aspects of such transformative scale-up include: relevance and responsiveness to community health needs; increasing the number of professional health workers and training institutions; and reforms in recruitment, teaching methods, and curricula in order to improve the quality and social accountability of graduates (8). Social accountability in this context is defined as ‘the obligation of medical schools to direct their education, research and service activities towards addressing the priority health concerns of the community, region, and /or nation they have the mandate to serve. The priority health concerns are to be identified jointly by governments, healthcare organisations, health professionals, and the public’ (9, p. 3). Social accountability is a key component of the World Health Organization's Global Toolkit for Evaluating Health Workforce Education (10). The toolkit proffers the six building blocks for transformative education as being: health workforce planning; governance, policy, and funding; national standards on accreditation, regulation, and vocational qualifications; curricula, faculty, and education; career and retention; and student selection (10).

There is a significant and diverse body of literature on nursing education, focusing *inter alia* on the following: professionalisation through the shift from a hospital-based apprenticeship model to baccalaureate nursing degrees in higher education institutions (11–22); nursing education theories (23); debates on competencies in pre-service training (17, 24–29); curriculum development for nurse educators (30–32); and student selection, experiences, and perceptions of the learning environment (33–38). In contrast to medical education where the notion of social accountability has put medical schools under the spotlight (39–43), there has been relatively little emphasis on the social accountability of nursing education institutions or their graduates. For example, there is no mention of social accountability in the 2009 global standards for the initial education of professional nurses and midwives (44). Although South Africa's 2012/13 Strategic Plan on Nursing Education, Training and Practice proposed that nursing education should be regarded as a national competence in order to enhance social accountability, this plan has not been implemented (45).

In South Africa, there are three categories of nurses: professional (registered) nurses with 4 years of training; enrolled nurses with 2 years of training, and nursing assistants or auxiliaries with 1 year of training. The majority of professional (registered) nurses are also midwives, and the terms ‘nurse’ and ‘midwife’ are used interchangeably in the Nursing Act (46). Nursing education is undergoing major changes, notably the requirement that a baccalaureate degree is a precondition for registration as a professional nurse, and the abolition of the enrolled nurse with 2 years of training in favour of a staff nurse with a 3-year college diploma (47). Nursing education takes place in a complex environment, which includes 20 out of 23 public universities, 12 public-sector nursing colleges (with numerous satellite training campuses) that are the responsibility of the nine provincial health departments, a nursing college run by the defence force, private nursing colleges run by the three major private hospital groups in South Africa, and private nursing schools that are run for profit (45, 48). This environment creates considerable fragmentation, and various layers of complexity. The public universities and public-sector nursing colleges are the only institutions that are allowed by law to offer the integrated course leading to registration as a professional nurse, which includes general, community health nursing, psychiatric nursing, and midwifery, through a 4-year degree or diploma, respectively (46). The South African Nursing Council (SANC) is the regulatory authority responsible for setting standards and accrediting nursing education institutions against those standards (46). The SANC does not require newly qualified professional (registered) nurses with 4 years of training to write a national licencing examination, but relies on the nursing education institution's own quality assurance systems to ensure an acceptable standard of education and competencies (46). However, the reality is that the quality of nursing education for registered nurses differs across the different educational institutions, and even from one nursing college to another managed by the same provincial health department (48). There are even more variations in the training of enrolled nurses (with 2 years of training) and auxiliary nurses (with 1 year of training), although these two categories of nurses are required to write a national licencing examination.

Given the global discourse on transforming health workforce education in support of universal health coverage (10), this paper uses a social accountability framework, specifically the six building blocks for transformative education, to explore key informants’ perspectives on nursing education in South Africa, in order to enhance the discourse on appropriate nursing education reforms and to make recommendations for policy implementation. The findings are part of a larger research project that examined casualisation of the nursing workforce (49, 50).

## Methods

The study was approved by the University of the Witwatersrand Human Research Ethics Committee (Medical). All participants received a study information sheet and gave informed written consent. Using a snowballing sampling technique, 44 key informants were selected purposively on the basis of their expertise or knowledge of the research area.

Semi-structured interviews were conducted with the key informants after informed consent had been obtained, using an interview guide developed specifically for the study by the research team and an advisory committee. In addition to questions on casualisation, the interview covered the following: achievements or progress by nurses or in nursing; challenges faced by nurses, both individually and by the nursing profession as a group; perceptions of changes in nursing in the 5 to 10 years preceding the interview; and recommendations for change. A member of the research team contacted each person identified as a potential key informant and requested an interview. If the person agreed to be interviewed, an appointment was set up for an interview at a place and time convenient for the informant. All interviews were conducted in English. Interviewers used probes to clarify responses and to obtain more detailed information.

Two researchers conducted the interviews between June and October 2009. Each key informant interview lasted about one hour, although the duration varied in some cases depending on the responses by informants. Interviews were recorded digitally, but interviewers also took detailed notes during the interview and wrote a synopsis of the interview. In some instances, respondents provided additional, relevant documentation to the interviewers.

The recorded interviews were transcribed verbatim. Data cleaning took 1 month and consisted of an iterative process of checking the transcribed interviews against the original recordings, correcting the text, checking the recordings again, and making final corrections. Prior to analysis, an audit of key informants was done, and each key informant was allocated a code number to ensure confidentiality of information. The coded interviews were consolidated into one file for ease of analysis.

The qualitative data were analysed using template analysis, which is a particular form of thematic content analysis (51). The WHO Global Toolkit for Evaluating Health Workforce Education was used as a template for the analysis of the key informant responses (10). The six building blocks – health workforce planning; governance policy and funding; national standards on accreditation, regulation, and vocational qualifications; curricula, faculty, and education; career and retention; and student selection – were used as *a priori* themes that were determined in advance of coding (51).

## Results

The 44 key informants were drawn from six categories: national and provincial government officials (*n=*16); executive nursing managers from the private sector or non-governmental organisations (*n=*10); nursing academics in universities or nursing colleges (*n=*8); representatives from the nursing council, nursing association, and/or health sector unions (*n=*5); and other health professionals, mainly doctors (*n=*5). Only two key informants from two health sector unions failed to respond to repeated requests for an interview.

The results on nursing education that emerged from the key informant interviews are presented according to the six building blocks for transformative health professional education (10).

### Health workforce planning

This building block measures the existence of information on the health status of the population or of individual subgroups as well as detailed information on health workforce demand and need, because these have an impact on health professional education (10). At the time of the study, there was a national Strategic Plan for Human Resources for Health (52), as well as a national Nursing Strategy (53). However, there were no national norms and standards for different categories of health workers, and several key informants commented on this gap, which has an impact on education and service delivery.

There are no staffing norms – it makes it difficult when opening new services – there needs to be broad consultation [on national norms]; it should be done in line with what is available [health workers] and link it up to the budget. (KI 14, provincial government manager, Gauteng)

I think that before we start to look at moonlighting the first thing we have to get [are] proper norms and standards … we are just working with what different people are saying and doing, but we haven't come up with proper staffing norms and standards for our own country and our own facilities … both in the private and public sectors. I am glad the Nursing Strategy is beginning to speak to that. (KI 16, private-sector nursing executive, Gauteng)

The staffing norms are not in place to say this is how we would like to staff either a primary health care centre or the medical ward or the general ward. The staffing norms are either outdated or unknown to the managers. (KI 35, human resource manager, international organisation)

### Governance, policy, and funding

This building block focuses on broad governance arrangements for health professional education (including collaboration across government ministries), policy development, and information on the funding of health workforce education (10).

A substantial number of key informants were of the opinion that the sub-optimal leadership provided by the Nursing Council as the regulator was the primary cause of many of the nursing problems experienced, as can be seen in the following excerpts:

Our Nursing Council is dysfunctional … we basically can't increase our numbers, open new centres, start new courses. You know we've tried to get permission to increase our numbers and to start satellite centres, while we wait for the new Regulations to be published, but we're just not getting there. (KI 1, private-sector nursing executive, Western Cape)

The last few [Nursing] Councils that we've had, we've really had problems with leadership. We were hoping that the new Council would be different, but when I listen to some of the things that people say, it's not different. It's about self-interest or self-serving interest rather than looking at the interest of the profession. So what is it that we doing wrong? I don't know. I mean how do we change that kind of culture? (KI 8, nurse educator)

The SANC was also criticised for the perceived delays in the implementation of the revised scopes of practice for the different nursing categories and the related training Regulations that guide nursing education institutions with regard to the preparation of these nurses. Key informants indicated that such delays stifle attempts to improve or expand nursing curricula and contribute to a state of inertia in the nursing education system, which had remained static instead of changing to meet the needs of the population or the health system. The Nursing Council was also criticised for its apparent reluctance to work with other government structures responsible for higher education.

The National Department of Health was also blamed by the key informants for much of the lack of planning and coordination as well as slow implementation, which hampers nursing education from moving forward.

I think there's a huge urgency to have leadership to take that on [an increase in nurse production] … both from the National [Department of Health] and also from the Nursing Council. And the fact that both institutions continue to be in disarray is worrying because we so need leadership and nobody else can really do that. (KI 7, human resource researcher, Western Cape)

The provincial health departments responsible for the implementation of nursing education in the public sector were also criticised for exacerbating fragmentation and contributing to a feeling of despondency among nurses:

The political people [in the provinces] don't talk to one another about health because they own the colleges. They [provinces] and higher education [Ministry] are not talking to one another. We don't get the feedback. So it's a major vacuum of uncertainty and everybody is now getting despondent. (KI 42, nursing academic, Free State)

Although policy development is important, the problems of health workforce production were exacerbated by insufficient resources in the public health sector and what some termed ‘transformation fatigue’ among nurses.

I think the biggest challenge that I have seen facing all nurses, regardless of whether you are in a rural area or the urban area – nurses in the public sector, I don't know about the private sector – is the frequency of the policy initiatives that contradict each other, and don't get seen through and don't have proper support and all of that; these lead to incredible transformation fatigue. And what I've seen happen is that nurses simply withdraw. (KI 7, human resource researcher, Western Cape)

Key informants were also of the opinion that there is lack of prioritisation of nursing, illustrated by the lack of funding for nursing strategic priorities. A senior provincial government manager commented as follows:

We keep on saying that nursing is the backbone of the health system, but when it comes to financing, it's difficult for them to finance nursing issues. For example, if we talk of training of nurse managers or the training of nurses, I have to go and knock on other people's doors and say, listen, I need to train nurses … but there is no specific budget. (KI 22, provincial government manager, Eastern Cape)

### National standards on accreditation, regulation, and vocational qualifications

This building block focuses *inter alia* on the need for accreditation, regulation, and quality control mechanisms for health workforce education in the country (10). In South Africa, the SANC is the regulatory authority for nursing education and practice and its mandate is prescribed by legislation (46). As indicated earlier, key informants commented on the sub-optimal functioning of the Nursing Council, which has an impact on the implementation of existing national standards, rather than the absence of accreditation.

The majority of key informants expressed concerns about the quality of teaching and learning, and there was some concern about the impending reforms in nursing education. In general, the medical doctors interviewed were of the opinion that the proposed reforms in nursing education have not been thought through, and one said the following:

I also do believe that training of [degree] nurses is not appropriate for what South Africa needs. I don't actually think we should be extending the university training. I think we should be extending the nursing colleges and training nurses for the primary function, that is, patient care and everything that goes around that. (KI 29, public-sector medical specialist, Gauteng)

The proposed Regulations also raised concerns among key informants about the lack of alignment between nursing training and the service or work environment. Some were of the opinion that nurses are inadequately prepared for the burden of disease that they face in hospitals or for working in a district health system.

### Curricula, faculty, and education

This building block focuses on appropriate curricula; community participation in health workforce education; the demographic profile of faculty and teaching staff; support for educators to engage in lifelong learning and continuing professional development; and community-based, interprofessional education based on principles of PHC and focusing on the social determinants of health (10).

Some of the key informants were of the opinion that nursing curricula are unresponsive to changes in disease burden and in the health system and that there is a disjuncture between government's emphasis on PHC and the training of nurses. These stated curriculum problems were exacerbated by the perception of the lack of skills among newly trained nurses, with many key informants pointing to skills gaps. These missing skills included inadequate social skills, lack of initiative, inability to apply theoretical knowledge to patient care, lack of basic nursing skills, and lack of understanding of professional practice.

Key informants also mentioned problems related to practical training, which included an insufficient number of good-quality practical training facilities, coordination of practical placements, and insufficient resources. A critical aspect mentioned was the lack of supervision and mentoring of students. One key informant said:

If I think back of our educational programmes, by the time you were a third-year nurse you would be in charge of the ward and run and manage the ward so well. We had mentors and we had people that we wanted to emulate … I don't think they [student nurses] have adequate mentors. They might be going out there to go and meet their clinical objectives but where are the mentors to [make sure] they achieve those clinical objectives? (KI 35, human resource manager, international organisation)

The key informants were critical of the quality of the nurse educators within many of the nursing education institutions, especially in the nursing colleges, who have not kept abreast of the practice environment and also lack modern teaching skills. There was a suggestion that because nurse educators have not kept abreast of these developments, they are not teaching up-to-date nursing procedures, which makes it difficult for the students to execute nursing tasks when they arrive in the hospitals. The shortages of nurse educators were seen as another significant challenge.

Really the training of nurse educators has been left behind. If we look at the standards, if we look at some of the resources in colleges, what the politicians expect … they [politicians] increase the [student] numbers by 100% but they don't provide the resources. (KI 40, nurse academic, North West)

The practical training is nearly non-existent and the supervision during training is scary and this is why they [nursing students] are not learning. They haven't got role models, they haven't got good teachers in the wards, and they are not learning how to do the job. The nurse educators are so busy trying to cope with these large groups now and they hardly ever get out into the hospitals, they themselves do not keep up to date, they are not even using up-to-date prescribed books or even South African-based prescribed books. (KI 20, provincial government manager, Gauteng)

### Career and retention

This building block focuses on the need to retain health workers particularly in underserved areas and to encourage their career development, as reflected in policies and plans and supportive measures in the workplace (10).

The majority of key informants pointed to resource constraints in nursing education institutions, particularly the lack of budgets, teaching infrastructure, student accommodation, and teaching equipment. Importantly, a similar lack of resources was experienced at the health facilities, which provide clinical training. Key informants pointed to the impact that resource constraints have on the quality of the nursing graduates. For example, in the public sector, they might not have exposure to new technology. In some instances, nursing education institutions based at universities have found creative alternatives, such as patient simulators. It was felt that the lack of resources has an impact on the retention of nurses, particularly in the public sector.

### Student selection

This building block focuses on student recruitment that is based on a combination of formal qualifications and transversal skills, reflective of underserved populations (10).

Key informants were very vocal about the problems of selection and suitability of applicants for entry into nursing. One said the following:

The other thing around [nursing] education is trying to get the right kind of nurse for this country. I think we put [too] much emphasis on the 4-year programme, whether a 4-year diploma or degree nurse – and they [nurses] get over-qualified and then they have aspirations to go all over. And what does the South African health-care system need in terms of the nurse? We really need to revisit the kind of nurse we need and what are the numbers we need to train. (KI 16, private-sector nursing executive, Gauteng)

There was a general perception that there is a problem in attracting nursing recruits who meet the selection criteria, because young people in South Africa have more career opportunities open to them. One commented as follows:

Nursing was a coveted job by young black women, so we used to get the best matriculants … so there was a time when we had quality people and they were in fact quite dedicated as well. Then I think over time there was an erosion of that, and it became a less attractive profession, poorly paid, long working hours, inflexible working hours, it lost its lustre and then young women looked for other career opportunities rather than doing nursing. (KI 29, medical specialist, Gauteng)

Key informants mentioned other factors that impact on student selection. These include the advanced age of students on commencement of training, which reduces the pool of nurses for further specialised training, and the relatively high attrition rate of students resulting in wastage of time and money in training them.

Almost one-third of the key informants were of the opinion that that the values and ethos of the current nursing students were different (and worse) compared with those held by nurses of the older generation and that the new generation was less caring towards patients and less committed to staying in nursing, as captured by the following quote:

I think the number one thing [problem] is the issue of the values in nursing, which perhaps may be different for different people but generally nursing was perceived to be a vocation for many of us – not the only choice of career. The values of caring for people and the importance of integrity and accountability were very important to us. (KI 19, retired nursing manager, Gauteng)

## Discussion

In South Africa, there is a well-established system of regulation and accreditation of nursing education through the SANC (46). This is commendable, as this is not the case in many low- and middle-income countries (3).

Using the six building blocks for transformative education and the voices of the 44 key informants, the study found sub-optimal governance on nursing education, thus confirming the findings of a policy analysis study that examined nursing education reforms in South Africa (47). The policy analysis study found slow progress, weak governance by the SANC and the Department of Health, and limited planning for implementation (47). A critical question that requires further research is why nurses do not demand greater accountability from the SANC and the Department of Health. The findings of the study underscore the importance of strategic leadership of key governance structures, such as the SANC. Such leadership will enable increased production of competent nurses, which will contribute to improved health system performance and ultimately to improved population health outcomes.

Key informants also pointed to problems with workforce planning, curricula, faculty and education, and student selection and training, which impact on nursing education. The issue of national staffing norms remains as important today as it was in 2009 when the interviews were conducted. Although South Africa has updated strategic plans on Human Resources for Health (54) as well as for Nursing Education, Training, and Practice (45), these do not contain detailed staffing norms. Staffing norms are not an end in themselves, but they provide guidance to managers and practitioners alike and assist with achieving equitable access to health-care providers, particularly in underserved urban and rural areas (55).

The comments from key informants suggest that curricula in nursing education institutions require revision so that they are more appropriate for the population and health system needs of South Africa. However, the lack of finalisation of the training Regulations and the scopes of practice was a major barrier to transformation of nursing education curricula. Although the training regulations were released at the end of 2012, the scopes of practice remain outstanding. The SANC continues to postpone the phasing-out of the legacy qualifications and, although the training of enrolled nursing auxiliaries and enrolled nurses will end in June 2015, a new date has been set for 2018 for the phasing-out of the programme leading to registration as a nurse (general, psychiatric, and community) and midwife. Hence, the implementation inertia described by some key informants continues. This inertia means that there has been little progress in implementing the 2009 global standards on nursing and midwifery education with regard to curriculum reform (44).

Nursing educator preparedness is another major issue that requires attention. Although key informants acknowledged the constraints within which nursing educators have to work, they were critical of the quality of the nurse educators. The views of key informants are borne out by the situation analysis of the 5-year Strategic Plan for Nursing Education, Training and Practice, which noted problems of insufficient numbers, heavy workloads, inadequate continuing professional development, outdated knowledge and skills, and an exodus from nursing education institutions due to the Occupation-Specific Dispensation, a public-sector financial incentive (45). Experience in other countries has shown that investment in nurse educators must accompany any major transformation in nursing education (11, 12, 31, 32). Although South Africa's Strategic Plan for Nursing Education, Training and Practice recommended the development of a national framework on nurse educators with dedicated resources (45), implementation has lagged behind, and many of the deadlines in the plan have passed already.

Key informants were of the opinion that the recruitment and selection of entrants to professional nursing accounted for some of the problems experienced in nursing. Another South African study also found that, although there are many more applicants to nursing courses than could be accommodated, they either did not meet the admission criteria, or nursing was the second or third choice (48). Nonetheless, the problems around student recruitment and selection to ensure that the best people enter the nursing profession are not unique to South Africa (21, 25–28, 37, 56).

The study findings suggest that nursing education in South Africa is grappling with getting the basics right for many of the building blocks considered essential for transformative education, with social accountability as a key feature. This paper makes an important contribution to the debate on issues that need to be addressed in the implementation of the impending nursing education reforms in South Africa, from the perspective of key opinion leaders in nursing and in the health sector. However, the findings are not generalisable, as the key informants were selected purposively. The information also represents the perceptions of key informants at a particular point in time, and hence we do not know how these perceptions have changed since the interviews were conducted in 2009. Nonetheless, it is one of the first studies using the WHO building blocks on transformative education and exploring the notion of social accountability as it relates to nursing education.

What then are the implications of the study findings for the implementation of the impending nursing education reforms in South Africa? The delays in the implementation of the proposed nursing education reforms provide a window of opportunity to incorporate the social accountability principles of responsiveness, relevance, quality, cost-effectiveness, and impact (9, 40, 42) into plans for implementation. Incorporating these principles requires stewardship and leadership from both the SANC and the Department of Health. The membership of SANC makes provision for community representatives, and the mandate of the Council is to protect the interests of the public. The SANC has the opportunity to embark on creative strategies to enhance community participation and increase public accountability.

The appointment of a Chief Nursing Officer at the beginning of 2014 is encouraging. We recommend that this incumbent draws on the skills and expertise of the nursing education structures, the national nursing association, student associations, and the many nurses who are keen to contribute to the current nursing education reform processes.

A great deal of research has been done on various factors relating to student selection, such as academic and non-academic predictors of success among student nurses (57), motives for entering nursing (35), and the role of gender (36). The selection criteria and system for student nurses into higher education should be based on the evidence already available at a global level, and efforts should be made to deal with its implementation considering the legal, sociocultural, political, and economic issues that will affect any decision taken.

Accountability for teaching and learning has to lie predominantly with the nursing education institutions themselves (43). The immediate way of institutionalising social accountability in the new system of nursing education is to ensure both that the curriculum is responsive to the needs of the community and that students are technically competent and graduate with the values and ethos of socially accountable graduates. Redman et al. addressed this issue many years ago by suggesting that a practice-oriented model of curriculum and competency assessment would address accountability requirements (37).

Programme assessment is equally important, as it provides evidence of educational effectiveness and therefore meets the demand for accountability (58). A third aspect of evaluation to improve accountability is raised by Thompson (59), who advocates widening the evaluation of individual faculty members to encompass assessment of the collective performance of the staff in a nursing education institution. This is because an individual lecturer cannot ensure that students complete the course as competent practitioners (59).

## Conclusion

Transformative education of nurses is critical in the move towards universal health coverage. Social accountability, which is an essential component of transformative education, necessitates that attention be paid to the issues of governance, curricula, nurse educator preparedness, student recruitment, and selection raised by key informants. Nursing education in South Africa is about to embark on profound changes with the move to the higher education sector. This transition provides a window of opportunity to address some of the issues that have troubled nursing education in the past, while embracing the concept of social accountability.
